# Evolution of Multidrug Resistance in Plasmodium falciparum: a Longitudinal Study of Genetic Resistance Markers in the Greater Mekong Subregion

**DOI:** 10.1128/AAC.01121-21

**Published:** 2021-11-17

**Authors:** Mallika Imwong, Kanokon Suwannasin, Suttipat Srisutham, Ranitha Vongpromek, Cholrawee Promnarate, Aungkana Saejeng, Aung Pyae Phyo, Stephane Proux, Tiengkham Pongvongsa, Nguon Chea, Olivo Miotto, Rupam Tripura, Chau Nguyen Hoang, Lek Dysoley, Nghia Ho Dang Trung, Thomas J. Peto, James J. Callery, Rob W. van der Pluijm, Chanaki Amaratunga, Mavuto Mukaka, Lorenz von Seidlein, Mayfong Mayxay, Nguyen Thanh Thuy-Nhien, Paul N. Newton, Nicholas P. J. Day, Elizabeth A. Ashley, Francois H. Nosten, Frank M. Smithuis, Mehul Dhorda, Nicholas J. White, Arjen M. Dondorp

**Affiliations:** a Department of Molecular Tropical Medicine and Genetics, Faculty of Tropical Medicine, Mahidol University, Bangkok, Thailand; b Mahidol–Oxford Tropical Medicine Research Unit, Faculty of Tropical Medicine, Mahidol University, Bangkok, Thailand; c Department of Clinical Microscopy, Faculty of Allied Health Sciences, Chulalongkorn University, Bangkok, Thailand; d Worldwide Antimalarial Resistance Network, Faculty of Tropical Medicine, Mahidol University, Bangkok, Thailand; e Division of Vector-Borne Diseases, Department of Disease Control, Ministry of Public Health, Nonthaburi, Thailand; f Myanmar Oxford Clinical Research Unit, Yangon, Myanmar; g Shoklo Malaria Research Unit, Faculty of Tropical Medicine, Mahidol University, Mae Sot, Thailand; h Savannakhet Provincial Health Department, Savannakhet, Lao People’s Democratic Republic; i National Center for Parasitology, Entomology, and Malaria Control, Phnom Penh, Cambodia; j Wellcome Sanger Institute, Hinxton, United Kingdom; k Centre for Tropical Medicine and Global Health, Nuffield Department of Medicine, University of Oxford, Oxford, United Kingdom; l Oxford University Clinical Research Unit, Hospital for Tropical Diseases, Ho Chi Minh City, Vietnam; m Institute of Research and Education Development, University of Health Sciences, Ministry of Health, Vientiane, Lao People’s Democratic Republic; n Lao–Oxford–Mahosot Hospital–Wellcome Trust Research Unit, Microbiology Laboratory, Mahosot Hospital, Vientiane, Lao People’s Democratic Republic

**Keywords:** *Plasmodium falciparum*, genetic resistance markers, Greater Mekong subregion

## Abstract

Increasing resistance in Plasmodium falciparum to artemisinins and their artemisinin combination therapy (ACT) partner drugs jeopardizes effective antimalarial treatment. Resistance is worst in the Greater Mekong subregion. Monitoring genetic markers of resistance can help to guide antimalarial therapy. Markers of resistance to artemisinins (*PfKelch* mutations), mefloquine (amplification of P. falciparum multidrug resistance-1 [*PfMDR1*]), and piperaquine (*PfPlasmepsin2/3* amplification and specific P. falciparum chloroquine resistance transporter [*PfCRT*] mutations) were assessed in 6,722 P. falciparum samples from Vietnam, Lao People’s Democratic Republic (PDR), Cambodia, Thailand, and Myanmar between 2007 and 2019. Against a high background prevalence of *PfKelch* mutations, *PfMDR1* and *PfPlasmepsin2/3* amplification closely followed regional drug pressures over time. *PfPlasmepsin2/3* amplification preceded piperaquine resistance-associated *PfCRT* mutations in Cambodia and reached a peak prevalence of 23/28 (82%) in 2015. This declined to 57/156 (38%) after first-line treatment was changed from dihydroartemisinin-piperaquine to artesunate-mefloquine (ASMQ) between 2014 and 2017. The frequency of *PfMDR1* amplification increased from 0/293 (0%) between 2012 and 2017 to 12/156 (8%) in 2019. Amplification of *PfMDR1* and *PfPlasmepsin2/3* in the same parasites was extremely rare (4/6,722 [0.06%]) and was dispersed over time. The mechanisms conferring mefloquine and piperaquine resistance may be counterbalancing. This supports the development of ASMQ plus piperaquine as a triple artemisinin combination therapy.

## INTRODUCTION

Early diagnosis and treatment, together with vector control, comprise the cornerstone of effective malaria control. Effective early treatment of Plasmodium falciparum infections prevents the progression of the disease to severe malaria, which still takes >400,000 lives every year (1). The first-line treatment of uncomplicated falciparum malaria in all countries where malaria is endemic is artemisinin combination therapy (ACT). New compounds are not expected to reach the market before 2026 (2). It is therefore of great concern to regional and global malaria elimination initiatives that artemisinin-resistant P. falciparum has emerged and spread in the Greater Mekong subregion (GMS) and, more recently, has emerged independently in Guyana, Papua New Guinea, Ethiopia, and Uganda, and particularly in Rwanda, where its prevalence has increased over recent years (3, 4). In infections with artemisinin-resistant P. falciparum, the malaria parasites are still cleared after ACT treatment, but because of the loss of ring-stage susceptibility, parasite killing is reduced and clearance is slower. As a result, the artemisinin component of the ACT contributes less to the antimalarial effect, and efficacy becomes more dependent on the partner drug. Currently, six ACTs are recommended: artesunate-sulfadoxine-pyrimethamine, artemether-lumefantrine (AL), artesunate-amodiaquine (ASAQ), artesunate-mefloquine (ASMQ), dihydroartemisinin-piperaquine (DHA-PPQ), and, most recently, artesunate-pyronaridine. When susceptibility to the partner drug declines, ACT efficacy drops significantly, and the proportion of recrudescent infections increases. This has been the pattern observed over the past decade in the GMS, where all six recommended artemisinin-based combination therapies have shown reduced efficacy at some point in time (5).

Increasingly, molecular genetic markers for antimalarial drug resistance have been identified, an advance that facilitates the monitoring of the emergence and spread of resistance. Currently, reliable molecular markers are available for P. falciparum resistance to artemisinins (mutations in the propeller region of *PfKelch*), sulfadoxine-pyrimethamine (mutations in the dihydrofolate reductase [*PfDHFR*] and dihydropteroate synthase [*PfDHPS*] genes), mefloquine (MQ) (amplification of the multidrug resistance-1 gene [*PfMDR1*]), and piperaquine (amplification of *PfPlasmepsin2/3* and specific mutations in the P. falciparum chloroquine resistance transporter gene [*PfCRT*]). Molecular markers accounting for the majority of the variance in susceptibility for the other partner drugs—lumefantrine, amodiaquine, and pyronaridine—are not well established. For some of the drugs, molecular markers can also monitor the evolution of increasing levels of resistance. For sulfadoxine-pyrimethamine, the sequential accumulation of mutations in DHPS and DHFR confer increasing levels of resistance against the two synergistic components (6, 7).

We describe here the evolution of markers of antimalarial drug resistance over time, and the observed combinations of antimalarial resistance markers, in a large set of P. falciparum samples obtained from the GMS countries, Vietnam, Lao People’s Democratic Republic (PDR), Cambodia, Thailand, and Myanmar, from 2007 to 2019. These observations provide additional insight into the development of mefloquine and piperaquine resistance in the region, and they show the very low prevalence of concomitant markers of resistance to both mefloquine and piperaquine. This has a direct impact on strategies for drug combinations and deployment.

## RESULTS

### *PfPlasmepsin2/3* and *PfMDR1* gene amplification and *PfKelch* mutations.

**FIG 1:**
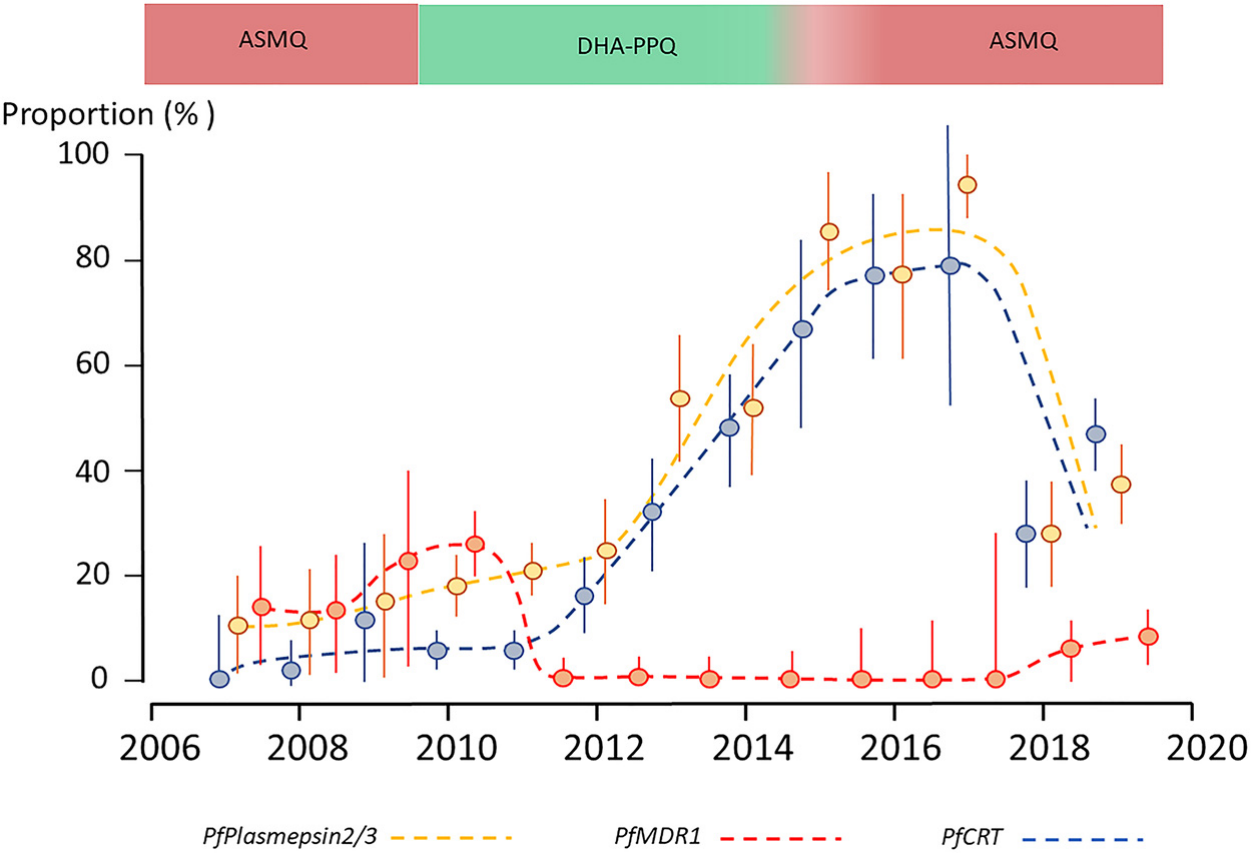
Changes in the frequencies of amplification of the *PfPlasmepsin2/3* and *PfMDR1* genes and in the frequency of novel piperaquine resistance-related *PfCRT* mutations during 2007–2019 in Cambodia. Error bars indicate 95% confidence intervals.

*PfPlasmepsin2/3* and *PfMDR1* copy numbers were assessed in 6,722 P. falciparum samples from the Greater Mekong subregion—Cambodia (*n* = 649), Laos (*n* = 1,332), Thailand (*n* = 666), Myanmar (*n* = 3,925), and Vietnam (*n* = 150)—between 2007 and 2019. *PfKelch* genotyping was performed for 3,848 of these samples; the remainder had insufficient parasite DNA left after the copy number assessments. Multiple copy numbers of *PfPlasmepsin2/3* were observed in 571 out of 6,722 (8.5%) samples, most of which (519/2,293 [22.6%]) were from the eastern GMS (Vietnam, Lao PDR, Cambodia, northeastern Thailand). The prevalence of multiple copy numbers was substantially lower in the western GMS (Myanmar and western Thailand): 52/4,429 (1.2%) (*P* = 0.00001). Amplification of the *PfMDR1* gene was observed in 321 of 6,722 (4.8%) samples, of which 78/2,293 (3.4%) were from the eastern GMS. Mutations in the propeller region of *PfKelch*, after amino acid position 440, were found in 2,116 of 3,848 (55.0%) samples, of which 555/761 (72.9%) were from the eastern GMS. In the eastern GMS, 432 of 555 *PfKelch* mutants (76.2%) carried the C580Y mutation. In Cambodia between 2007 and 2017, 68 of the 69 parasites with *PfPlasmepsin2/3* amplified (99%) also carried a *PfKelch* propeller region mutation; after the switch to ASMQ as the first-line treatment, this proportion decreased to 61/88 (69%) (Fig. 1).

The prevalence of *PfMDR1* and *PfPlasmepsin2/3* amplification in each country over time was associated with the concurrent first-line antimalarial drug treatment in those countries. In Myanmar, where the first-line therapy is AL, the prevalence of *PfMDR1* (144/3,925 [3.7%]) and *PfPlasmepsin2/3* (50/3,925 [1.3%]) amplification remained low. In Cambodia, the prevalence of *PfMDR1* amplification during the period of first-line treatment with ASMQ reached 16/85 (19%) (Fig. 1), but parasites with *PfMDR1* amplified disappeared after ASMQ was replaced with DHA-PPQ. After the deployment of DHA-PPQ, the prevalence of parasites with *PfPlasmepsin2/3* amplified increased to 23/28 (82%) in 2015. After the slow transition back to ASMQ, starting in 2014, this proportion declined to 57/156 (37%) in 2019. The prevalence of parasites carrying multiple copy numbers of *PfMDR1* increased from 0/293 (0%) between 2012 and 2017 but after full redeployment of ASMQ in 2017 was back to 12/156 (8%) in 2019.

**FIG 2:**
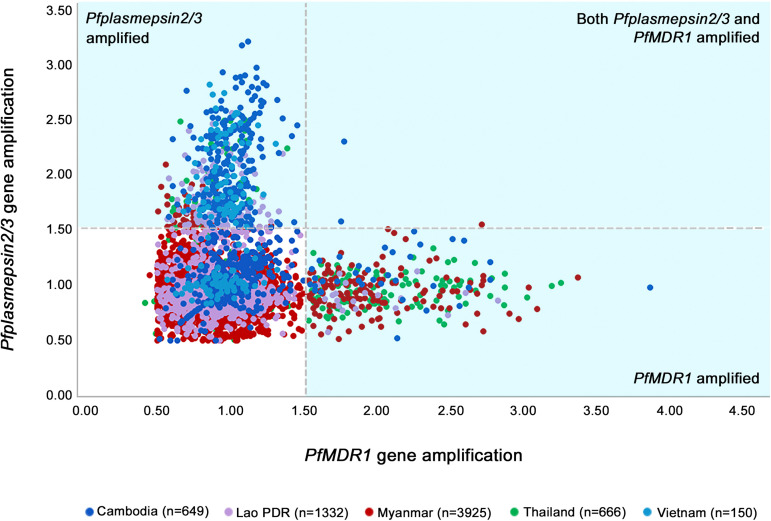
Distribution of *PfPlasmepsin2/3* and *PfMDR1* copy number estimates in 6,722 P. falciparum samples obtained from Greater Mekong subregion countries between 2007 and 2019, color-coded according to country. The shaded areas represent *PfPlasmepsin2/3* and *PfMDR1* estimates with indeterminate results, defined as a <90% chance of representing a single copy number versus multiple copy numbers of the gene.

By use of the conventional cutoff of 1.5 to denote gene amplification, parasites with concomitant amplification of *PfPlasmepsin2/3* and *PfMDR1* were very rare (Fig. 2). Such concomitant amplification was observed in only four isolates (4/6,722 [0.06%]): one sample from Pailin, Cambodia, collected in the year 2008 (1.58 and 1.76 *PfPlasmepsin2/3* and *PfMDR1* copies, respectively), one sample from Pursat, Cambodia, collected in 2019 (2.30 and 1.78 *PfPlasmepsin2/3* and *PfMDR1* copies, respectively), and another two from Kayin State, Myanmar (2/6,722 [0.02%]), collected in 2016 (1.51 and 1.55 *PfPlasmepsin2/3* copies and 2.08 and 2.72 *PfMDR1* copies). Application of the stricter cutoff value, corresponding to a 90% chance of a real amplification of these genes, reduced the number of samples with concomitant multiple copy numbers of *PfPlasmepsin2/3* and *PfMDR1* to three parasite samples (0.04%), collected in 2008, 2016, and 2019.

### *PfCRT* mutations in relation to *PfPlasmepsin2/3* and *PfMDR1* amplification.

A total of 536 P. falciparum isolates, collected between 2007 and 2019 from Cambodia (*n* = 478) and Vietnam (*n* = 58), were tested for the *PfCRT* mutations associated with piperaquine resistance. *PfCRT* mutations were found at positions T93S (12.7% [68/536]), H97Y/L (23.5% [126/536]), F145I (6.0% [32/536]), I218F (4.9% [26/536]), M343I/L (2.6% [14/536]), and G353V (5% [27/536]). In addition, the CVIET haplotype without any other *PfCRT* mutation was observed in 208/537 (38.81%) parasites; the CVIDT haplotype without other mutations was found in 47/536 (8.77%); and the *CVMNK* haplotype without other mutations was found in 1/536 (0.2%). Double mutations of *PfCRT* (not including the CVIET, CVIDT, and CVMNK haplotypes) were not observed, except in 13 samples with multiple clone infections (see Fig. S2 to S4 in the supplemental material).

The prevalence of novel piperaquine resistance-associated *PfCRT* mutations increased over time (Fig. 1). In 2007, 11% (5/42) of P. falciparum samples showed *PfPlasmepsin2/3* gene amplification, whereas no parasites had one of the novel piperaquine resistance-associated *PfCRT* mutations. After that, the prevalence of *PfPlasmepsin2/3* amplification increased to 25/100 (25.0%) in 2012 and 9/9 (100%) in 2017, together with an increase in novel *PfCRT* mutations to 16/100 (16.0%) (8 H97Y/L, 6 I218F, 1 M343I/L, and 1 G353V mutation) in 2012 and 7/9 (78%) (3 T93S, 2 H97Y/L, and 2 I218F mutations) in 2017. There was a strong association between *PfPlasmepsin2/3* amplification and the presence of the downstream *PfCRT* mutations (*r*^2^ =0.89). Of the 293 parasites carrying one of the novel *PfCRT* mutations, 216 (73.7%) also carried multiple copy numbers of *PfPlasmepsin2/3*.

**FIG 3:**
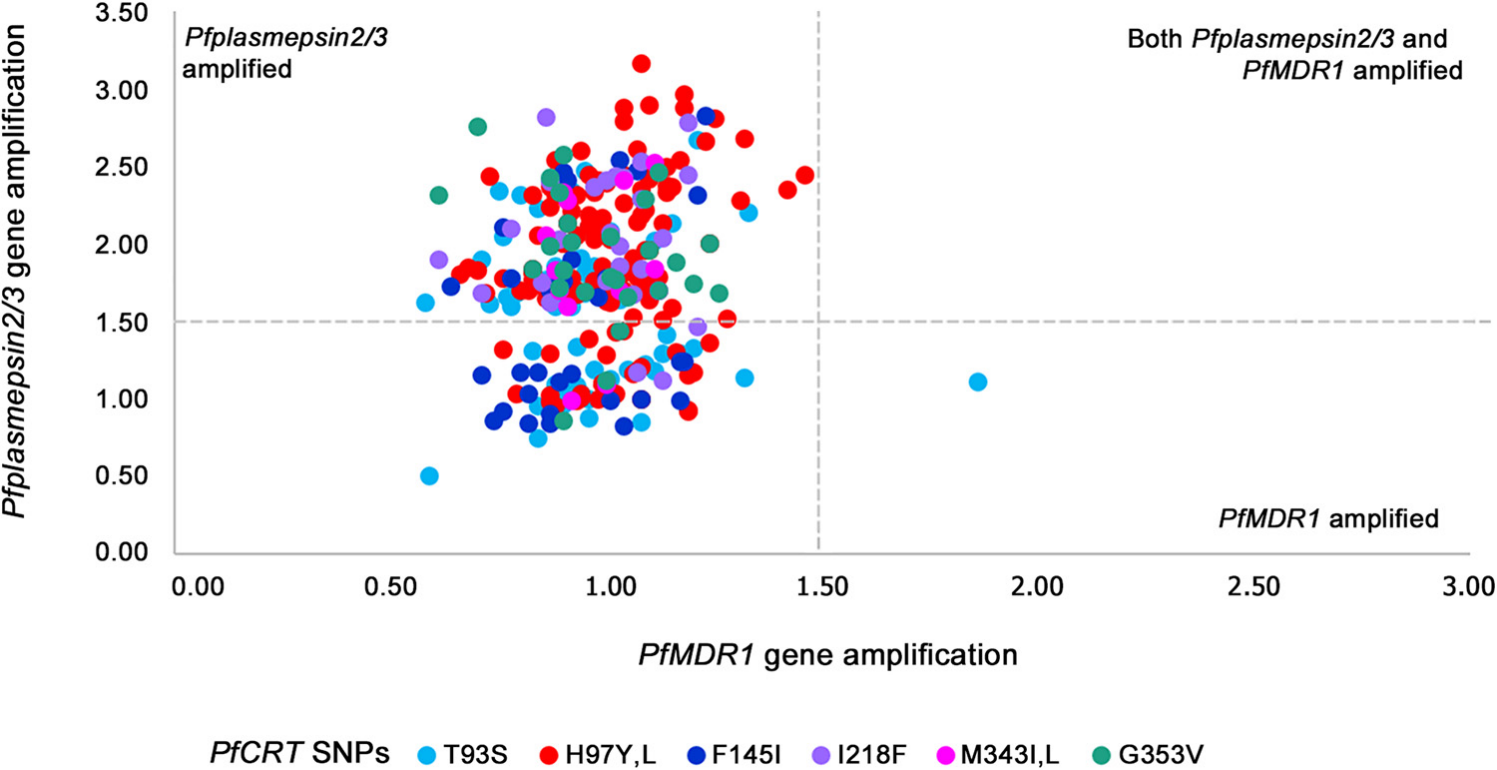
Relation between *PfMDR1* amplification, *PfPlasmepsin2/3* amplification, and novel piperaquine resistance-associated *PfCRT* mutations.

Among 478 samples with complete data, we observed only a single sample (0.21% [1/478]), collected in the year 2018 from northern Cambodia, harboring a piperaquine resistance-associated *PfCRT* mutation together with amplification of *PfMDR1* (Fig. 3). This sample showed a single copy of *PfPlasmepsin2/3*. In this study, we did not identify a single sample showing a combination of all four resistance genes (amplification of *PfKelch*, *PfPlasmepsin2/3*, and *PfMDR1* and a piperaquine resistance-associated *PfCRT* mutation) (Fig. 3).

## DISCUSSION

This study in the GMS analyzed the temporal trends in the prevalence of molecular markers for mefloquine resistance (*PfMDR1* amplification) and piperaquine resistance (*PfPlasmepsin2/3* amplification and novel piperaquine resistance-associated *PfCRT* mutations). Since most resistance mechanisms result in a fitness disadvantage, the prevalence of resistant parasites and their evolution with the spread of increasingly fit lineages depend on the drug pressures on the parasite population. The results of this molecular epidemiology study thus need to be interpreted in the contexts of the use of different drugs in different countries and changes over time. Cambodia introduced ASMQ as the first-line antimalarial treatment in 2000, then changed to DHA-PPQ in 2010, and subsequently gradually changed back to ASMQ from 2014 to 2017. Vietnam deployed ASMQ until 2005 and then moved to DHA-PPQ until 2020, when four provinces changed to artesunate-pyronaridine; another two provinces did so in 2021. In Myanmar and Lao PDR, AL remains the first-line treatment, with limited use of DHA-PPQ in Myanmar. Thailand used ASMQ as its first line treatment until 2015, when it changed to DHA-PPQ. As with adjacent Cambodia, northeastern Thailand switched back to ASMQ in 2019.

In Myanmar and Lao PDR, with low levels or no drug pressure from either mefloquine or piperaquine, the prevalence of parasites carrying either amplified *PfMDR1* or *PfPlasmepsin2/3* remained very low in this study. In western Thailand and Cambodia, the prevalence of multiple *PfMDR1* copy numbers increased significantly during the deployment of ASMQ and was associated with high failure rates in patients with combined artemisinin- and mefloquine-resistant infections (8). In Cambodia, the prevalence of multiple *PfMDR1* copy numbers rapidly declined after the change in first-line therapy toward DHA-PPQ in 2010. This has been confirmed in several studies (9–12) and is explained mainly by the fitness costs associated with *PfMDR1* gene amplification in the absence of mefloquine drug pressure (13). This has also been shown in *in vitro* cultures (14). In Cambodia and Vietnam, amplification of *PfPlasmepsin2/3* increased with the deployment of DHA-PPQ and increased much more rapidly in parallel with the increasingly high treatment failure rates with DHA-PPQ observed since 2013 (15, 16). In addition to *PfPlasmepsin2/3* amplification, novel mutations in the *PfCRT* gene, in addition to the chloroquine resistance-related K76T mutation (in the CVIET, CVIDT, and CVMNK haplotypes), have been associated with piperaquine resistance. These novel “downstream” mutations are closely linked to, and were preceded by, amplification of *PfPlasmepsin2/3*, as shown in Fig. 1. Among patients with P. falciparum infections carrying multiple *PfPlasmepsin2/3* copies and one of the PfCRT H97Y, F145I, and G353V mutations, treatment failure rates were higher after treatment with DHA-PPQ than for infections with parasites with only *PfPlasmepsin2/3* amplified (13). The role of *PfPlasmepsin2/3* amplification in piperaquine resistance is still uncertain. *Ex vivo* drug sensitivity testing of parasites with *PfPlasmepsin2/3* amplified showed increased resistance to piperaquine in a bespoke piperaquine survival assay (17). However, gene-edited P. falciparum parasites with multiple *PfPlasmepsin2/3* copies and overexpression of *Pf*Plasmepsin2 did not show increased piperaquine resistance (18). In contrast, gene-edited parasites with *PfCRT* H97Y, F145I, M343L, or G353V mutations are resistant to piperaquine *in vitro* (19). *PfPlasmepsin2/3* amplification could still play an indirect role in piperaquine resistance, but this is currently unclear. The strong selective sweep of a single *PfKelch* C580Y mutation-containing P. falciparum lineage in the eastern GMS under DHA-PPQ drug pressure was likely initially driven by artemisinin resistance. The predominance of this lineage increased rapidly after 2009 in countries deploying DHA-PPQ, and it acquired *PfPlasmepsin2/3* amplification and, only more recently, the novel *PfCRT* mutations (20). This sequence of events is also supported by a detailed genomic epidemiological study from the same area using whole-genome sequencing, showing the initial spread of a P. falciparum colineage with a *PfKelch* C580Y mutation and *PfPlasmepsin2/3* amplification, which then diversified and acquired one of the novel *PfCRT* mutations (21). All studies thus far found that the novel piperaquine-associated single nucleotide polymorphisms (SNPs) in *PfCRT* are mutually exclusive.

Both artemisinin resistance and piperaquine resistance contributed to the high DHA-PPQ failure rates in Cambodia and Vietnam. Piperaquine resistance alone is associated with recrudescence rates of approximately 20% assessed 42 days after treatment with DHA-PPQ, compared to 45% in P. falciparum infections with both artemisinin and piperaquine resistance (12). Recrudescent resistant infections are overall more transmissible than other infections, which drives their spread (22). Artemisinin-resistant infections have high rates of gametocytemia and may be more transmissible than other infections even before treatment failure rates begin to rise (23). After the withdrawal of DHA-PPQ in Cambodia in 2016, the prevalence of parasites with *PfPlasmepsin2/3* amplified declined from close to 100% to 38%, but these parasites have not disappeared, in contrast with the complete disappearance of *PfMDR1* amplification after mefloquine withdrawal. This may suggest either a lower fitness cost for parasites carrying multiple *PfPlasmepsin2/3* copy numbers or some other, unidentified advantage not associated with piperaquine resistance. The relative fitness of P. falciparum parasites carrying multiple copies of *PfPlasmepsin* or the novel *PfCRT* mutations has not been established. Alternatively, the currently very low level of multiplicity of malaria infection may allow relatively unfit parasites to persist in the absence of competition. Concomitant *PfMDR1* amplification and *PfCRT* mutations associated with piperaquine resistance were not observed, except in one parasite strain with a single *PfPlasmepsin2/3* copy number. Parasites carrying both an amplified *PfMDR1* gene and an amplified *PfPlasmepsin2/3* gene were also very rare; only single cases were observed in 2008, 2016, and 2019, for an overall prevalence of 0.06% (4/6,722), or 0.04% (3/6,722) when the stricter cutoff value is applied. Assuming free mixing between parasite populations, a rough estimate of the total expected parasite strains with both the *PfPlasmepsin2/3* and *PfMDR1* genes amplified would be around 17, a number obtained by simply multiplying the proportions of each amplified gene by year and the total number of parasite samples assessed per year. Also, in Cambodia between 2014 and 2016, when both ASMQ and DHA-PPQ were deployed, not a single parasite carrying amplification of both genes was observed. The observations suggest that amplification of both genes in the same P. falciparum parasite may confer a fitness disadvantage or compromised transmissibility. Interestingly, the increase in *PfMDR1* gene amplification in Cambodia after the redeployment of ASMQ has been less pronounced than in the first decade of the millennium, when deployment of this ACT in Cambodia followed nearly 2 decades of mefloquine monotherapy in adjacent Thailand. Despite the increasing use of ASMQ since 2014, with full deployment since 2017, the prevalence of parasites with multiple *PfMDR1* copies increased to only 8% (12/160) in 2019, and ASMQ remains to date an effective treatment for uncomplicated falciparum malaria in Cambodia (3). The much lower levels of transmission, and thus the lower level of competition, may again be a contributor. It may also be that amplification of both *PfMDR1* and *PfPlasmepsin2/3* within the same parasite renders the parasite very unfit. The continued relatively high prevalence of *PfPlasmepsin* amplification would then be a barrier to a rapid increase in *PfMDR1* amplification and thus to the reemergence of mefloquine resistance.

These data support the continued deployment of ASMQ in Cambodia. The very low prevalence of concomitant mefloquine and piperaquine resistance also supports the strategy of combining both drugs in triple artemisinin combination therapies (TACT), in which an artemisinin derivative is combined with two well-matched existing partner drugs (5). This provides a more effective treatment for multidrug-resistant falciparum malaria but could also extend the life span of existing antimalarial drugs by slowing or preventing the emergence of resistance. The TACT DHA-PPQ plus MQ was recently studied in a large randomized trial in uncomplicated falciparum malaria (24). This TACT was shown to be well tolerated, safe, and highly effective, including in areas of multidrug-resistant malaria such as Cambodia and Vietnam. TACT could therefore be one of the few remaining treatment options in the GMS. However, sustained efficacy will depend on the absence of fit parasites which are resistant to both partner drugs, since these parasites would be readily selected with the deployment of a TACT containing DHA-PPQ plus MQ.

Other studies confirm the absence of concomitant *PfPlasmepsin2/3* and *PfMDR1* amplification (19, 25). However, a retrospective study in Cambodia in 2017 reported a much higher prevalence than in this study; as many as 30% of parasites had amplification of both *PfMDR1* and *PfPlasmepsin2/3*, although this was not associated with increased rates of treatment failure with ASMQ (26). We believe that the different methodology used to assess gene copy numbers in this study, dye-based quantitative PCR (qPCR) assays, might have resulted in substantial overestimation of gene amplifications. Although dye-based qPCR assays, including those with EvaGreen and SYBR green, have certain advantages over probe-based qPCR assays in terms of cost-effectiveness and time efficiency, these dyes can bind nonspecifically to double-stranded DNA outside the targeted qPCR product. This causes increased background signal and false-positive results (27). In the current study, in parasites with a copy number readout above 1.52, the statistically determined cutoff for a >90% chance of genuine *PfPlasmepsin2/3* amplification, the proportion of parasites carrying the characteristic SNP at the duplication breakpoint for *PfPlasmepsin2/3* was 88%. This confirms the reliability of our results but also shows that in a minority (around 10%) of parasite samples, *PfPlasmepsin2/3* amplification might have been assigned wrongly.

A shortcoming of our study is that gene amplification in a minor P. falciparum clone in patients with multiple clone infections might not have been detected by using the current cutoff for the estimated copy number. This cannot be a large confounder, since multiple clone infections were identified in only 263 of 2,710 samples (9.7%). Another caveat is that the absence of *PfMDR1* amplification might not exclude mefloquine resistance, since the drug is thought to have several targets (28). In earlier studies of mefloquine resistance in Thailand, *PfMDR1* amplification accounted for only two-thirds of the variance in susceptibility (13). Concomitant resistance to piperaquine and mefloquine might then not be detected by the current resistance markers. This emphasizes the importance of continuing to test *in vitro* drug susceptibility—particularly in treatment failures.

In conclusion, our study shows that the molecular genetic markers for mefloquine and piperaquine resistance have evolved differently in the western and eastern GMS. This appears to have resulted from the differences in antimalarial drug pressure. In contrast to the disappearance of *PfMDR1* amplification after the discontinuation of ASMQ, the prevalence of *PfPlasmepsin2/3* amplification in Cambodia remains high after the discontinuation of DHA-piperaquine. Concomitant amplification of *PfMDR1* and *PfPlasmepsin2/3* in the same P. falciparum parasite, as well as simultaneous occurrence of the novel *PfCRT* mutations and *PfMDR1* amplification, is extremely rare. Mechanisms conferring mefloquine and piperaquine resistance may counteract each other. This can be evaluated in the laboratory with gene-edited P. falciparum strains and through continued genetic epidemiological surveillance. These results provide support for the development and evaluation of a TACT containing artesunate, mefloquine, and piperaquine.

## MATERIALS AND METHODS

### Sample collection and processing.

As part of studies on the treatment, epidemiology, and targeted elimination of artemisinin-resistant malaria (ClinicalTrials registration no. NCT01350856, NCT02453308, NCT03384498, NCT03355664, and NCT01872702), venous blood samples, filter paper blood spots, and completed rapid diagnostic test strips were collected from patients with microscopy- or rapid-test-confirmed uncomplicated falciparum malaria, as well as from healthy individuals in villages where targeted malaria elimination activities were planned. The study sites in Myanmar, Thailand, Cambodia, Lao PDR, and Vietnam were subjects of large multinational observational and treatment studies in patients with falciparum malaria (TRAC I and TRAC II), or of large-scale malaria prevalence surveillance as part of malaria elimination studies. Full details of these clinical and epidemiological studies have been published previously, and some of the raw data used for this study is included in these publications (9, 20, 23, 24, 29, 30). Approvals for the studies were obtained from the Ethical Review Boards of the Faculty of Tropical Medicine, Mahidol University (MUTM 2017-045-03, MUTM 2011-015-01) and the University of Oxford Tropical Medicine Ethics Committee (protocols 527-17, 06-11, 1017-13, 1015-13, 32-17), the Department of Medical Research, Ministry of Health (Myanmar), the Lao National Ethics Committee for Health Research, and the National Ethical Committee for Health Research in Cambodia.

DNA was extracted from dried blood spots, completed rapid diagnostic test strips (both stored desiccated at room temperature), and frozen whole-blood samples by standard methods at the Faculty of Tropical Medicine, Mahidol University, Bangkok, Thailand. DNA was purified using a Qiagen kit (Qiagen, Germany) according to the manufacturer’s instructions.

### Assessment of mutations in *PfKelch* and *PfCRT*.

Polymorphisms in the *PfKelch* gene were examined by nested PCR amplification covering the propeller region of the gene as described previously (23), followed by sequencing of the gene using an ABI sequencer (Macrogen Inc, South Korea). The sequencing results were then aligned against the *PfKelch* gene (PF13_0238) of the reference strain 3D7 (NCBI reference sequence no. XM_001350122.1). Analysis was performed with BioEdit software (Abbott, CA, USA).

*PfCRT* was amplified from the DNA template using nested PCR. A PCR-restriction fragment length polymorphism assay was developed to assess *PfCRT* mutations related to piperaquine resistance identified in a previous study (19). These included the following single nucleotide polymorphisms (SNPs): N88K, T93S, H97Y, F145I, I218F, CVMNK72–76CVIET, N326S, M343L, G353V, I356T, and R371I. Digestion fragments were analyzed on a 3% agarose gel. For quality control, a random one-third of all PCR products were sent for DNA sequencing at Macrogen Inc, South Korea.

### Assessment of *PfPlasmepsin2/3* and *PfMDR1* gene amplification.

*PfPlasmepsin2/3* and *PfMDR1* copy numbers were quantified using relative quantitative real-time PCR (TaqMan real-time PCR) on a Corbett Rotor-Gene Q system (Corbett Research, Australia). The primers and probes have been described previously (10, 13). Amplification was performed in triplicate on a total volume of 10 μl as multiplex PCR using a QuantiTect Multiplex PCR NoROX kit (Qiagen, Germany). Copy number estimates were calculated as 2^–ΔΔ^*^CT^*, where ΔΔ*C_T_* denotes the difference between the change in the threshold cycle (Δ*C_T_*) of the unknown sample and the Δ*C_T_* of the reference sample. Reactions were repeated whenever the profile did not conform to exponential kinetics, or if the standard deviation of the ΔΔ*C_T_* values was >1.5 or the *C_T_* value of the PCR was >35. To confirm amplification and to resolve indeterminate results, samples passing these criteria but with an estimated copy number of >1.3 were also retested once, and the last result counted as final. For the main analysis, a cutoff copy number estimate of 1.5 was used to distinguish single- from multiple-copy *PfPlasmepsin2/3* and *PfMDR1* gene carriage, as used in previous studies (31–33). In addition, we defined the cutoffs for the 90% probabilities that the copy number estimate denotes a single copy number rather than multiple copy numbers of the gene. These probabilities were based on the distributions of the results obtained by the formula calculating the estimated copy number (see Fig. S1 in the supplemental material). This approach acknowledges that values around the cutoff will include the tail ends of the distributions of copy number estimate values representing P. falciparum samples carrying one versus two copies of the *PfPlasmepsin2/3* or *PfMDR1* gene. For this assessment of adapted cutoff values, samples carrying multiple P. falciparum clones were excluded. Using this approach, values between 1.14 and 1.52 for *PfPlasmepsin2/3* and values between 1.15 and 1.61 for *PfMDR1* were considered indeterminate, i.e., the distinction of single from multiple copy numbers of the gene was uncertain (Fig. S1). For 2,710 samples, *PfPlasmepsin2/3* amplification was also assessed by genotyping the SNP characteristic of the duplication breakpoint (34). Among parasites with a copy number estimate above the 90% probability cutoff of 1.52, *PfPlasmepsin2/3* amplification was confirmed in 595/675 (88%) using this alternative method. For estimates of <1.14, the 90% probability cutoff for a single gene copy number, and for indeterminate values between 1.14 and 1.52, the proportions of parasites with *PfPlasmepsin2/3* amplified according to the presence of the breakpoint SNP were 75/1,734 (4%) and 114/301 (38%), respectively.

### Availability of data and materials.

All data generated or analyzed during this study are included in this published article and its supplemental material files.
