# Supplementary material for: Evolution of Multidrug Resistance in Plasmodium falciparum: a Longitudinal Study of Genetic Resistance Markers in the Greater Mekong Subregion
Source: Antimicrob Agents Chemother. 2021 Nov 17;65(12):e01121-21. doi: 10.1128/AAC.01121-21 (PMC8597770; doi:10.1128/AAC.01121-21)
Supplement: Supplemental file 1 — Fig. S1 to S4. Download aac.01121-21-s0001.pdf, PDF file, 0.5 MB [file aac.01121-21-s0001.pdf]

Evolution of multidrug resistance in *Plasmodium falciparum* in the Greater Mekong Subregion: a longitudinal study of genetic resistance markers

Supplementary material

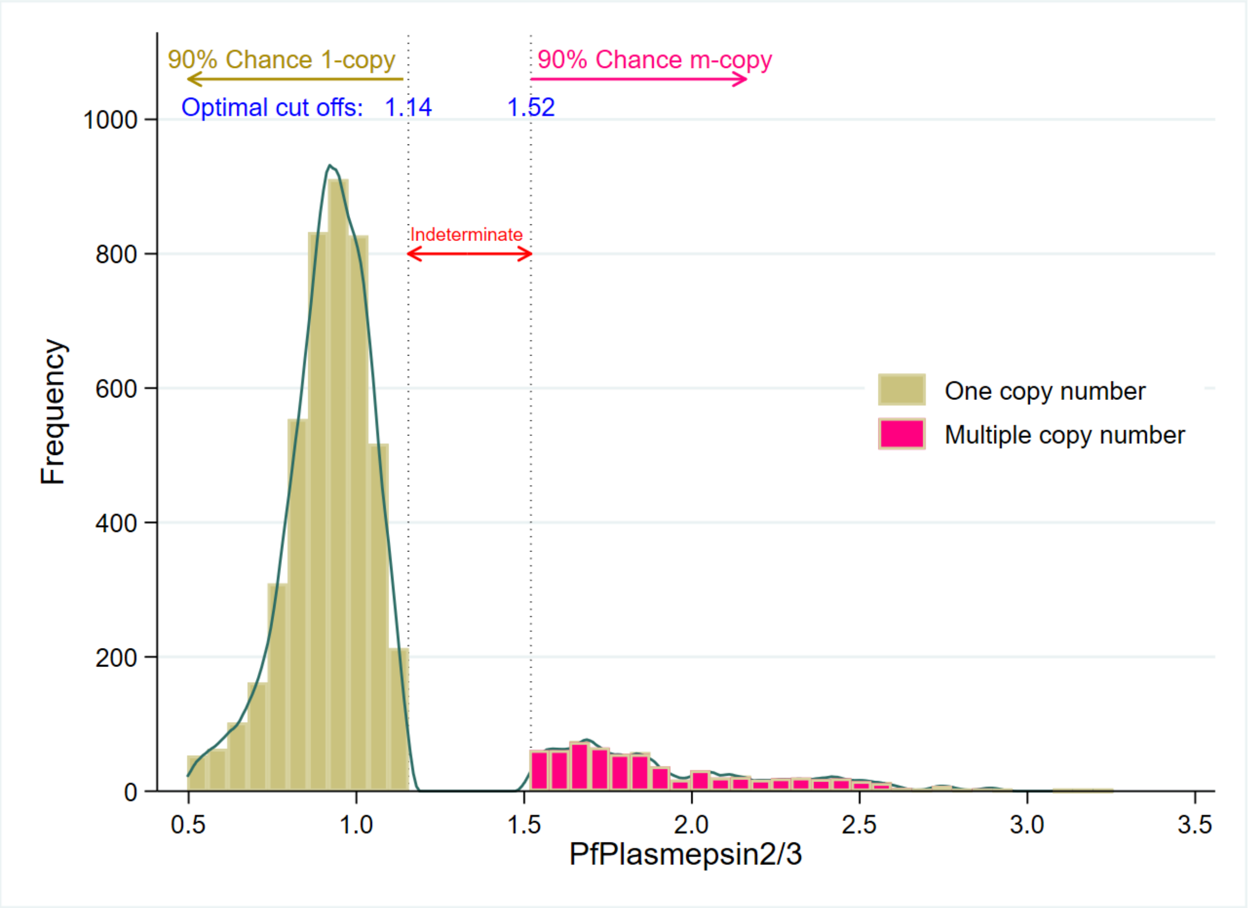

Supplementary figure 1a. A histogram of the distributions of the calculated PfPlasmepsin2/3 copy number values showing distinct groups as defined by cluster analysis.

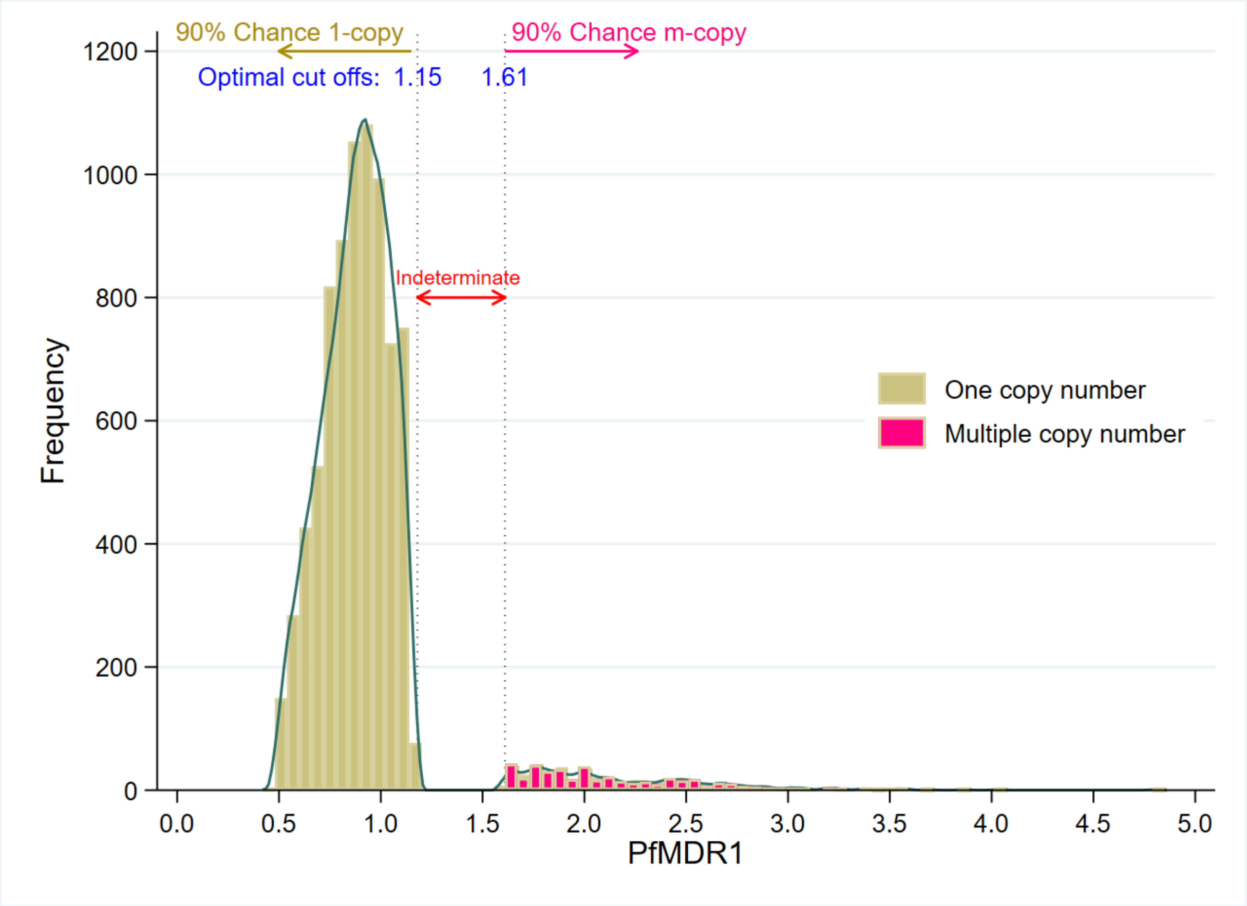

Supplementary figure 1b. A histogram of the distributions of the calculated PfMDR1 copy number values showing distinct groups as defined by cluster analysis.

Statistical cluster analysis was used to determine the optimal cut-offs to distinguish between the distributions denoting multiple versus a single gene copy number[1,2,3,4]. Visual inspection of the histograms identified 2 main contributing distributions for each of the figures. Cluster analysis using a 2-means algorithm then identified the means and distributions of the contributing clusters. From these, the 90th percentile cut-off for the single-copy cumulative density distribution, and the 10th percentile for the cut-off for the multiple-copy distribution were determined, using Stata version 16.

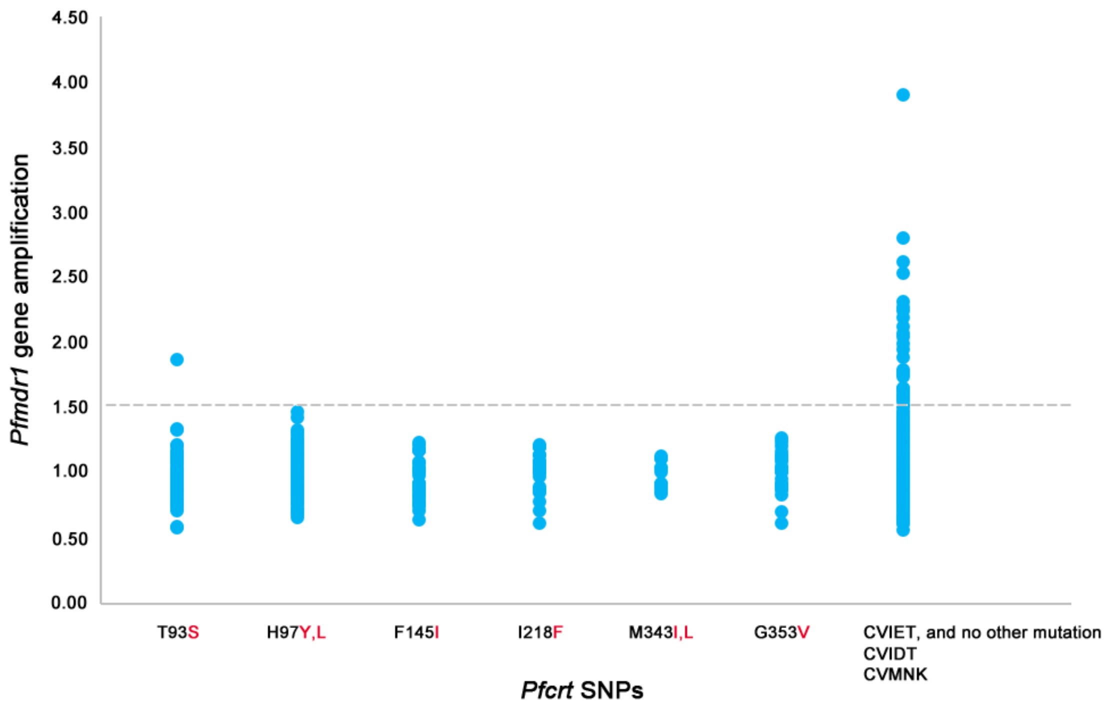

Supplementary figure 2. Presence of piperaquine resistance associated PfCRT mutations versus the presence of PfMDR1 gene amplification. Samples were collected from Cambodia (n=478) and Vietnam (n=58) between 2007 and 2019.

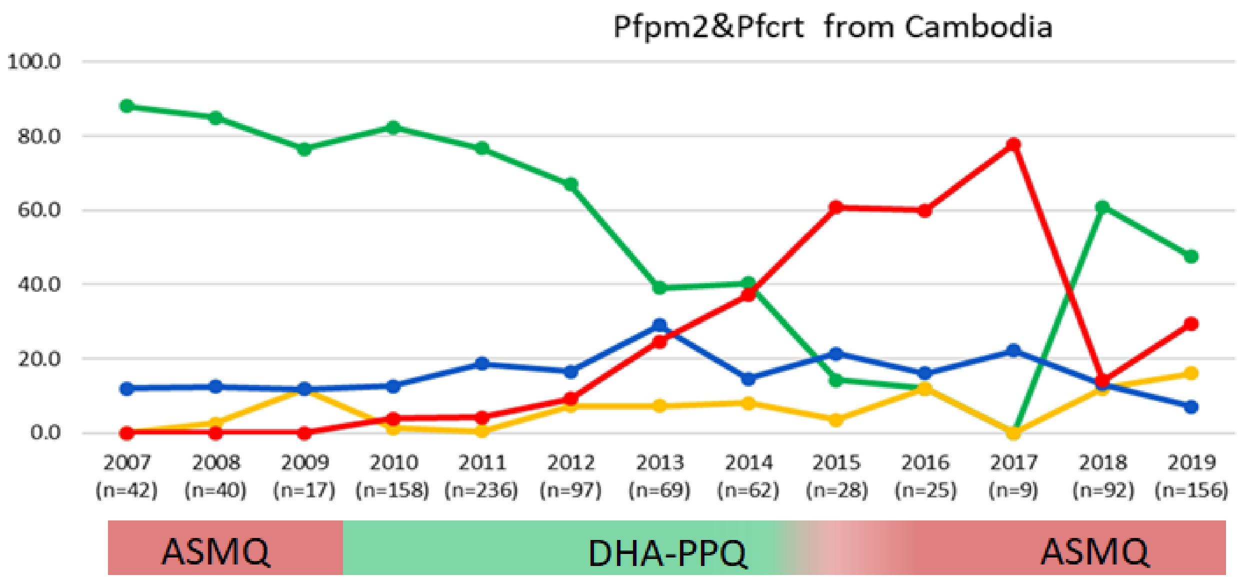

Supplementary figure 3. Prevalence over time of parasites carrying different combinations of *PfPlasmepsin2/3* copy number and piperaquine associated *PfCRT* mutation at positions 93, 97, 145, 218, 343 or 353. Samples were obtained from Cambodia from 2007 to 2019.

Single Pm2+No mutation CRT (93,97,145,218,343,353)

Single Pm2+ Mutation CRT (93,97,145,218,343,353)

Multiple Pm2+No mutation CRT (93,97,145,218,343,353)

Multiple Pm2+ Mutation CRT (93,97,145,218,343,353)

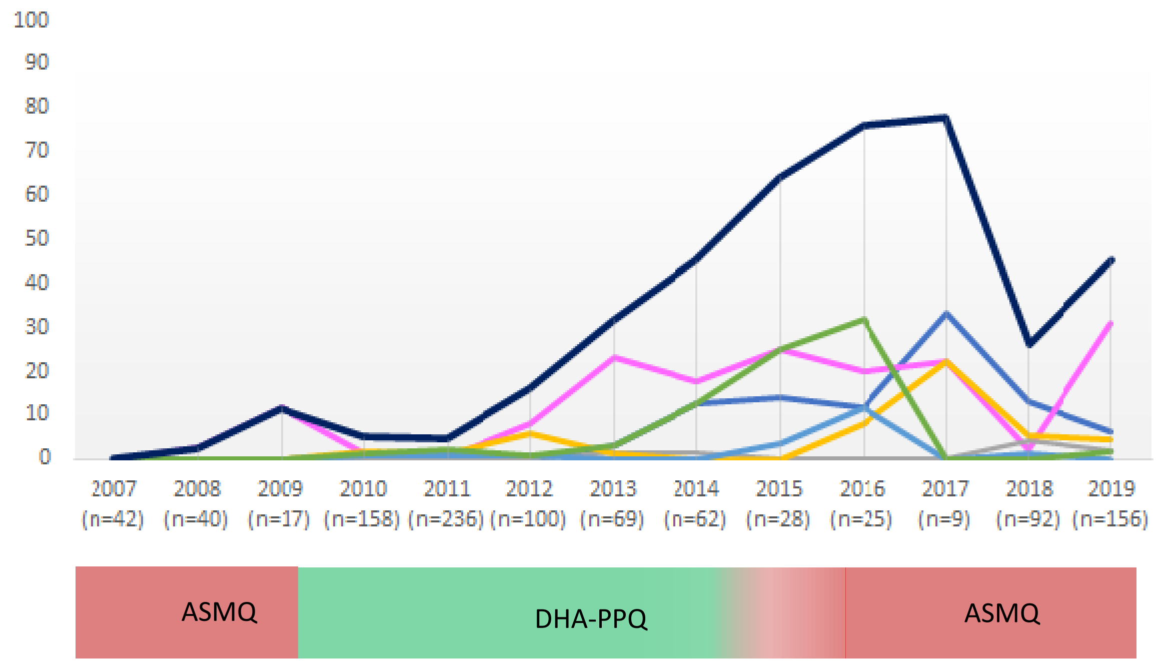

Supplementary figure 4 Prevalence of piperaquine resistance associated *PfCRT* mutations in Cambodia from 2007 to 2019

T93S H97Y/L F145I I218F M343I/L G353V % Total mutations of Pfcrt (93,97,145,218,343,353)

References

1. Hatamlou, Abdolreza. "In search of optimal centroids on data clustering using a binary search algorithm." *Pattern Recognition Letters* 33.13 (2012): 1756-1760. Reference Link

2. Li, Jinhua, et al. "Robust k-median and k-means clustering algorithms for incomplete data." *Mathematical Problems in Engineering* 2016 (2016). Reference Link

3. Silitonga, Parasian. "Clustering of Patient Disease Data by Using K-Means Clustering." *International Journal of Computer Science and Information Security (IJCSIS)* 15.7 (2017): 219-221. Reference Link

4. Makles, Anna. "Stata tip 110: How to get the optimal k-means cluster solution." *The Stata Journal* 12.2 (2012): 347-351. Reference Link

5. Shrestha B et al., "Distribution and temporal dynamics of *P. falciparum* chloroquine resistance transporter mutations associated with piperaquine resistance in Northern Cambodia". *J. Infect. Dis*. 2021 (PMID:33528566) https://pubmed.ncbi.nlm.nih.gov/33528566/
